# New Record of *Encarsia protransvena* and Confirmed Occurrence of *Encarsia hispida* (Hymenoptera: Aphelinidae) as Parasitoids of *Singhiella simplex* (Hemiptera: Aleyrodidae) in Italy

**DOI:** 10.3390/insects16010040

**Published:** 2025-01-03

**Authors:** Giuliano Cerasa, Luigi Tomasello, Gianluca Melone, Elia Russo, Gaetano Siscaro, Carmelo Cavallaro, Annamaria Ienco, Francesca Laudani, Vincenzo Palmeri, Orlando Campolo, Francesca Garganese, Francesco Porcelli, Paolo A. Pedata, Vittorio Farina, Giovanni Gugliuzza, Roberto Rizzo, Stefania Laudonia, Gabriella Lo Verde

**Affiliations:** 1 Department of Agricultural, Food and Forestry Science, University of Palermo (UNIPA), Viale delle Scienze, Ed. 5, 90128 Palermo, Italy; giuliano.cerasa@unipa.it (G.C.); ltomasello51@gmail.com (L.T.); vittorio.farina@unipa.it (V.F.); 2 National Research Council, Institute for Sustainable Plant Protection, P. le E. Fermi 1, 80055 Portici, Italy; gianluca.melone1998@gmail.com (G.M.); paolo.pedata@ipsp.cnr.it (P.A.P.); 3 Department of Agricultural Sciences, University of Naples Federico II (UNINA), Via Università 100, 80055 Portici, Italy; elia.russo@unina.it; 4 Department of Agriculture, Food and Environment, University of Catania (UNICT), Via Santa Sofia, 100, 95123 Catania, Italy; gaetano.siscaro@unict.it (G.S.); carmelo.cavallaro@unict.it (C.C.); 5 Department of Agriculture, Mediterranean University of Reggio Calabria (UNIRC), Loc Feo di Vito, 89100 Reggio Calabria, Italy; annamaria.ienco@unirc.it (A.I.); francesca.laudani@unirc.it (F.L.); vpalmeri@unirc.it (V.P.); orlando.campolo@unirc.it (O.C.); 6 Department of Soil Sciences, Plants and Food, University of Bari Aldo Moro (UNIBA), Via Amendola 165/A, 70126 Bari, Italy; francesca.garganese@uniba.it (F.G.); francesco.porcelli@uniba.it (F.P.); 7 CREA-Research Centre for Plant Protection and Certification, c/o Department of Agricultural, Food and Forestry Science, University of Palermo (UNIPA), Viale delle Scienze, Ed. 5, 90128 Palermo, Italy; giovanni.gugliuzza@crea.gov.it (G.G.); roberto.rizzo@crea.gov.it (R.R.)

**Keywords:** fig whitefly, natural enemies, aphelinid wasps, morphological and molecular identification

## Abstract

*Encarsia protransvena* (Hymenoptera: Aphelinidae) is recorded here for the first time in Italy as a parasitoid of the whitefly *Singhiella simplex* (Hemiptera: Aleyrodidae), one of the most invasive alien pests of exotic *Ficus* species. *Singhiella simplex,* originating from the Oriental region, has established a global presence. Monitoring of the whitefly and its parasitoids was conducted in the southern areas of Italy, providing crucial insights into their distribution and interactions. The taxonomic identity of *E. protransvena*, was confirmed by scrutiny of morphological and molecular taxonomic characters. At the time of writing, we also obtained some specimens of *Encarsia hispida* from the host collected in Campania. We reserve the right to provide bio-ethological information on the species in the future. Comprehensive illustrations and diagnostic features are provided for the host and the parasitoids. An identification key is included for all *Encarsia* species associated with *S. simplex*, which provides a valuable tool to distinguish these aphelinid wasps for future research and applications in biological control programs.

## Simple Summary

We report the detection of *Encarsia protransvena* and confirm *Encarsia hispida* as parasitoids of the exotic invasive whitefly *Singhiella simplex* (Hemiptera: Aleyrodidae) in Italy. Native to the Oriental region, *S. simplex* has now spread worldwide, causing significant damage to exotic fig plants in southern Italy, providing a unique opportunity to study its natural interactions. The whitefly and its parasitoids were collected in some south Italian regions, obtaining two species of Chalcidoidea Aphelinidae. Using a combination of morphological and molecular analyses, we identified *E. protransvena*, a species reported as the only known natural enemy in the Mediterranean region. This discovery increases our understanding of the biology of both the pest and its parasitoid, offering valuable insights for future biological control programs. As we write this note, we also have obtained specimens of *Encarsia hispida* from the host collected in Campania. We aim to provide bio-ethological information about the species in the future.

## 1. Introduction

The fig whitefly *Singhiella simplex* (Singh, 1931) (Hemiptera: Aleyrodidae) is an invasive sap-feeding pest native to Southeast Asia that primarily infests ornamental plants of the genus *Ficus*, mainly *Ficus benjamina* L. and *Ficus microcarpa* L.f., but can also target lesser host plant species, such as *Hibiscus* spp. (Malvaceae) and *Mangifera indica* L. (Anacardiaceae) [1,2,3,4,5,6].

After its first record as an invasive species in the United States (Hodges, 2007), the pest spread to Central and South America and the Caribbean [5]. In 2014, the fig whitefly was found in Europe on several fig species in Cyprus [4] after its European recording, *S. simplex* spread across several Mediterranean countries [7,8,9,10,11,12,13], including Italy, where it has significantly impacted urban trees in roadsides, parks, and gardens [14,15,16,17]. Due to the significant phytosanitary risks associated with *S. simplex*, the pest was included in the EPPO Alert List from 2014 to 2018 [2].

Recent studies on the biology of *S. simplex* indicate that, under favourable climatic conditions such as those in Mediterranean countries, this whitefly can develop several overlapping generations per year [18].

The direct damage caused by *S. simplex* is mainly due to sap-sucking by the nymphs, which gradually weakens and yellows the infested leaves. Unlike most whitefly species, *S. simplex* feeds on both sides of the leaf [19]. It excretes considerable honeydew, promoting intense sooty mold that extensively coats the host plant. Infestations cause extensive defoliation and twig dieback, posing an additional threat to the overall plant health [6,7].

Several natural enemies of *S. simplex* have been reported in the literature, including predators and entomopathogens [6,20,21,22,23,24]. Different species of hymenopteran parasitoids belonging to the genus *Encarsia* Förster (Hymenoptera: Aphelinidae) are present worldwide and associated with the whiteflies, among them the worldwide species *Trialeurodes vaporariorum* (Westwood) [25] and *Aleurocanthus spiniferus* (Quaintance, 1903), recently introduced in Europe [26]. Three species of hymenopteran parasitoids in the genus *Encarsia* are reported as associated with the fig whitefly: *Encarsia singhiellae* Shih and Polaszek, 2015; *E. hispida* De Santis, 1948 and *E. protransvena* Viggiani, 1985 [5,27,28,29]. The recording of *E. tricolor* Förster, 1878 [30] has been considered a misidentification based on the aphelinid known species distribution [5]. The platigastrid *Amitus bennetti* Viggiani and Evans, 1992, and the eulophid *Baeoentedon balios* Wang, Huang and Polaszek, 2014, have also been reported [28,31]. Occasionally, males of *E. variegata* Howard, 1908, develop as hyperparasitoids on the eulophid previously mentioned [6,28]. *Encarsia protransvena* is the only confirmed parasitoid of *S. simplex* in the Mediterranean region [24]. At the same time, the recent report of *E. hispida* [17] deserves confirmation. In particular, the iconography reported in the article does not show morphological characteristics of *E. hispida* at all. A confirmation has come while we write this note, thanks to a recent collection of *S. simplex* in the Campania region with parasitization activity of *E. hispida*, currently in reduced numbers, contextual with that due to *E. protransvena*.

In 2024, surveys in southern and central Italy (Apulia, Basilicata, Calabria, Campania, Lazio, and Sicily regions) monitored the presence of the invasive *S. simplex*, and female parasitoids belonging to the genus *Encarsia* were detected in multi-site collections of the whitefly.

Here, we combined morphological analysis and molecular data from mitochondrial and nuclear markers to confirm the taxonomic identity of *E. protransvena* and *E. hispida.* We also documented the distribution of *E. protransvena* in Italy and provided a detailed morphological characterization to improve its identification. Finally, we propose an identification key for all *Encarsia* species associated with *S. simplex*, which will aid in distinguishing these aphelinid wasps in the following research and biocontrol programs management.

## 2. Materials and Methods

### 2.1. Hosts and Parasitoids Collection

Surveys have been conducted on *F. microcarpa* and *F. benjamina* trees planted in urban greeneries, parks, and roadsides in Sicily (Provinces of Palermo and Catania), Calabria (Province of Reggio Calabria), Apulia (Bari, metropolitan city), and on isolated plants of private gardens in Lazio (Province of Latina), in Campania (Provinces of Naples, Caserta and Salerno), and in Basilicata (Province of Matera). The collecting sites in which the whitefly was present are listed in Table 1. During each collection, infested leaves with *S. simplex* immature stages were gathered and then examined by a stereoscopic microscope in the laboratory. The collected material was isolated in small rearing cages and kept under laboratory conditions (26° C, 70% RH) until the whitefly or the parasitoid adult emergence. The whitefly nymphs and adults of both the host and the parasitoid were preserved in ethanol (EtOH 90%, *V*/*V* water). Adults of whitefly and parasitoids and leaves bearing the whitefly instars were photographed using a Wild-Heerbrugg M8 stereoscopic microscope equipped with a digital camera (Canon 7D, Canon Inc., Tokyo, Japan). Single montage images were obtained from image stacks with the freeware CombineZP [32] and then processed in Adobe Photoshop CS4.

### 2.2. Morphological Identification

Whitefly slide mounting followed standard methods reported by Martin [33] and Pizza and Porcelli [34], whereas slide mounting of parasitoids was carried out according to Noyes [35] and Polaszek et al. [36].

Mounted insects were examined in a bright field on a Zeiss Universal Photomicroscope III and photographed using a Leica DFC420C camera mounted on a Leica DM 2005 compound microscope (Leica Application Suite software, Leica Microsystems Ltd., Buccinasco (MI), Italy). CombineZP [32] produced image stacks post-processed in Adobe Photoshop CS4.

Identification of both adult and pupae of the whitefly was carried out according to Jensen [37], Hodges [30], Suh et al. [38], and Kondo and Evans [19]. The parasitoid identifications were carried out following the original description by Viggiani [39] and the identification keys reported by Heraty and Polaszek [40] and Polaszek et al. [41]. The specimens of *Encarsia* collected in different areas (see Examined material) and prepared on slides were compared with type material deposited in the Silvestri Museum of the University of Naples collection.

### 2.3. Molecular Identification

Sequencing of the parasitoid specimens emerged from *S. simplex* (see Examined material) targeted a portion of the mitochondrial cytochrome *c* oxidase subunit 1 (*COI*) gene and the nuclear D2 expansion segment of 28S (*28S-D2*) rDNA. These molecular markers were selected for their wide application in the taxonomy of Chalcidoidea [42,43,44]. Newly emerged parasitoids were individually killed in 96% ethanol, and genomic DNA was extracted using the non-destructive Chelex-proteinase K protocol by Gebiola et al. [45], preserving the morphological details of specimens.

The *COI* segment was amplified with the forward primers C1-J-2183 or C1-J-2195 paired with the reverse TL2-N-3014 [46], previously used for molecular characterization of *Encarsia* species [43,44]. The *28S-D2* region was amplified using the universal primers D2F and D2R developed by Campbell et al. [47]. Table 2 lists the oligonucleotide sequences and their characteristics. The polymerase chain reaction (PCR) runs were performed using DreamTaq Green PCR Master Mix (Thermo Fisher Scientific, Ferentino (FR), Italy) in a total volume of 25 µL, comprising 12.5 µL PCR Master Mix (2X), 2 µL DNA template, 1 µL of each primer (10 µM), and 8.5 µL nuclease-free water. Thermal cycling conditions included an initial denaturation at 95 °C for 3 min, followed by 40 cycles of 95 °C for 30″, 50–55 °C (depending on melting temperatures of the primer) for 30″, and 72 °C for 1′. The protocol concluded with a final extension at 72 °C for 7 min. PCR products were separated on a 1% agarose gel stained with SYBR™ Safe DNA Gel Stain (Thermo Fisher Scientific) and observed under a ChemiDoc (Bio-Rad, Segrate (MI), Italy) transilluminator. Positive amplicons were sent to Eurofins Genomics (Ebersberg, Germany) for purification and bi-directional sequencing.

Forward and reverse reads were manually edited using BioEdit version 7.2.5 [48] (Hall, 1999) to correct overlapping peaks in the electropherograms. Low-quality bases were trimmed from both ends, and consensus sequences were generated. The *COI* sequences were virtually translated into protein using the Transeq (EMBOSS) tool to check for frameshifts or premature stop codons. All sequences were submitted to NCBI’s BLASTn protocol in GenBank to verify matches against standard databases using default parameters.

## 3. Results

### 3.1. Morphological Identification

**Figure 1:**
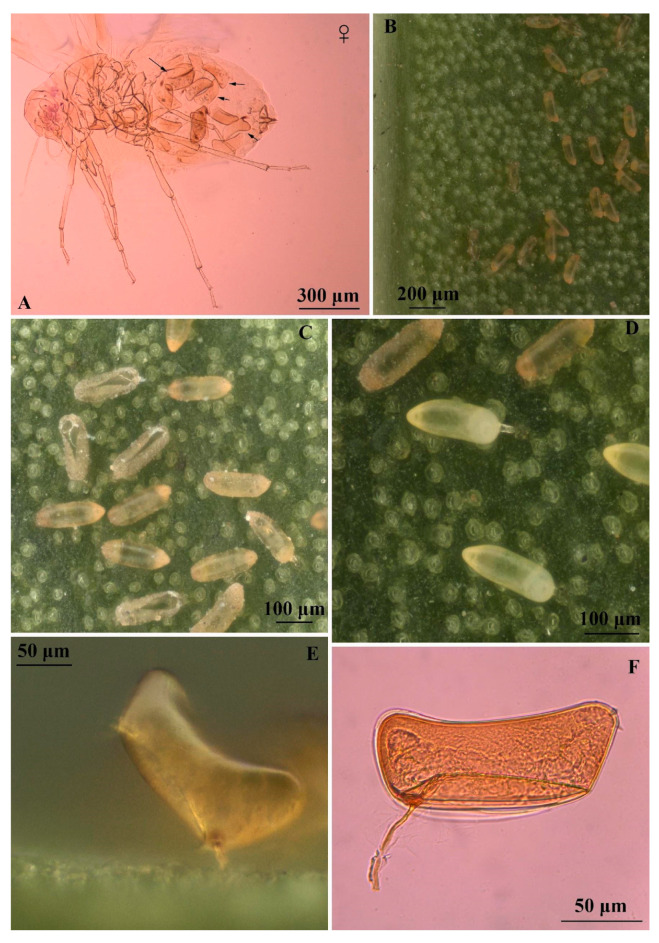
*Singhiella simplex*: (**A**) slide-mounted adult female, arrows show eggs retained in the abdomen; (**B**–**F**) whitefly eggs.

**Figure 2:**
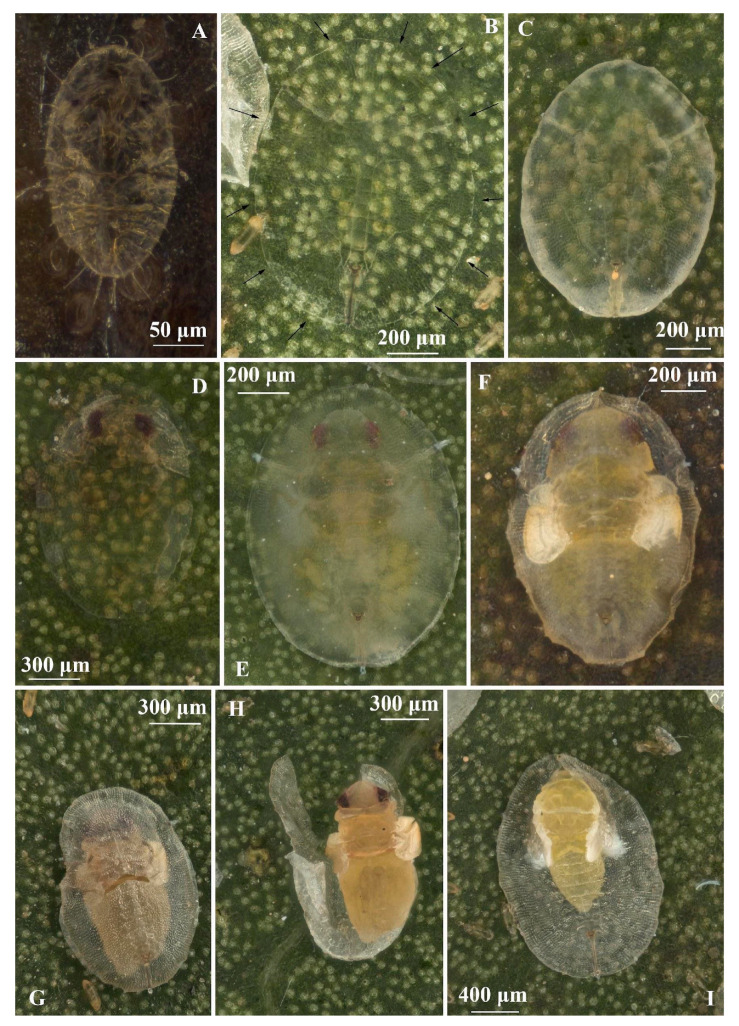
Immature stages of *Singhiella simplex*: (**A**) whitefly first instar; (**B**–**E**) second to fourth instar; (**F**–**I**) prepupa and pupa.

**Figure 3:**
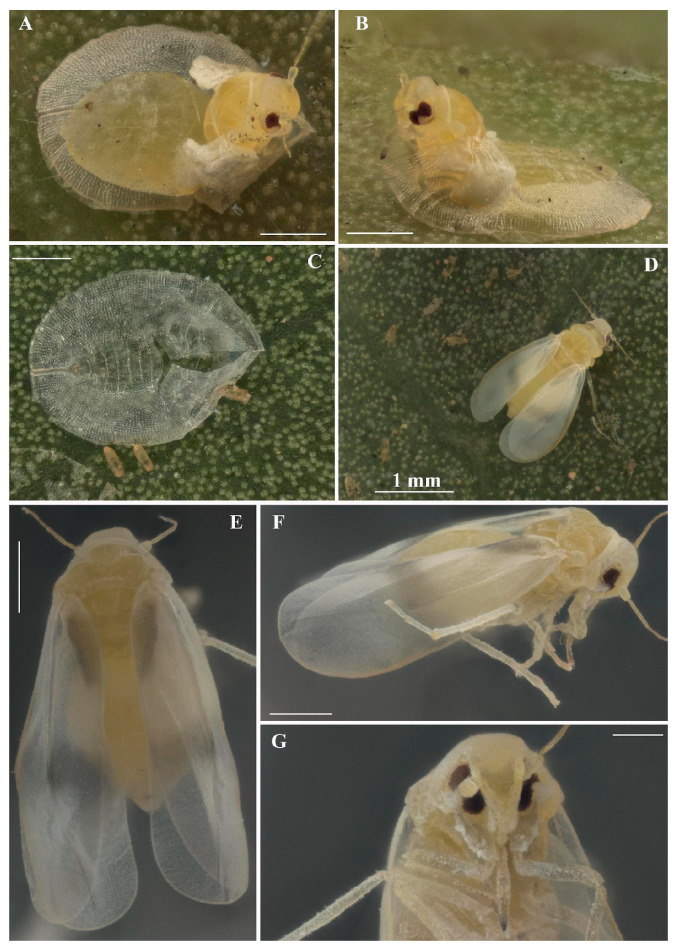
*Singhiella simplex*: (**A**,**B**) emerging adults; (**C**) exuvia (pupal case); (**D**–**G**) adults in dorsal, lateral and ventral view (scale bar: 300 µm, except in (**D**)).

**Figure 4:**
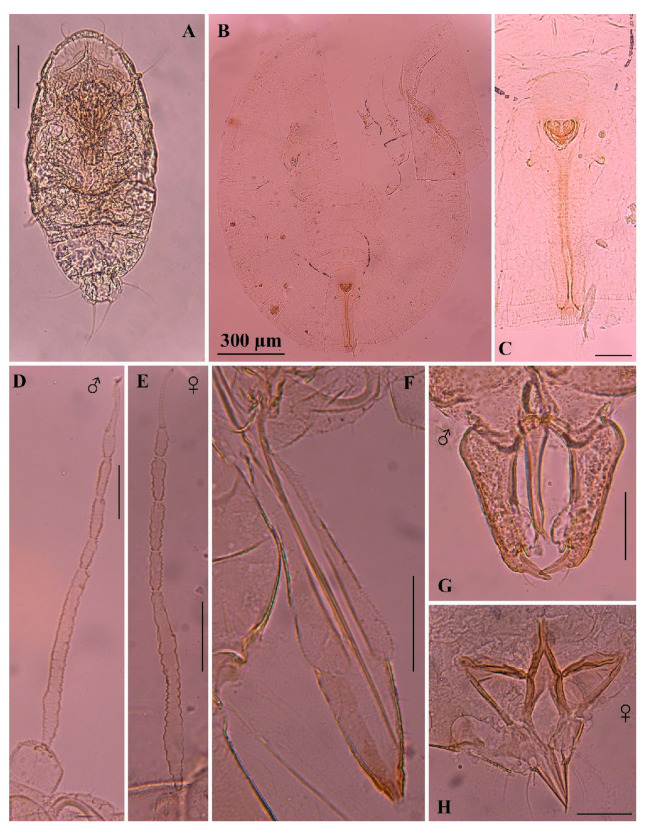
*Singhiella simplex*, microscope slide mounted: (**A**) first instar; (**B**,**C**) pupal case and enlargement shows vasiform orifice and caudal furrow; (**D**) antenna of male; (**E**) antenna of female; (**F**) needle-like mouthparts; (**G**) genital capsule, claspers and aedeagus of the male; (**H**) female genitalia (scale bar: 50 µm, except in (**B**)).

The identity of *S. simplex* was confirmed based on the morphology of both adults and immature instars (Figure 1, Figure 2, Figure 3 and Figure 4). The insect was found in all sampled sites only in Sicily and Calabria. In Apulia, the whitefly was recorded only in two out of six sites, where *Macrohomotoma gladiata* Kuwayama, 1908 (Hemiptera: Psylloidea: Homotomidae), another fig pest introduced in the EPPO area [48], was always present. Finally, no *S. simplex* was found in the only sampling site in the Basilicata region (Policoro, Matera province).

Parasitoids were obtained from all sampled sites where the whitefly was found, except the Apulia sites. Based on morphological features, the emerged parasitoid species were identified as *E. protransvena* and *E. hispida* (Hymenoptera: Aphelinidae). Their taxonomy, host association, and distribution are briefly discussed below.

**Figure 5:**
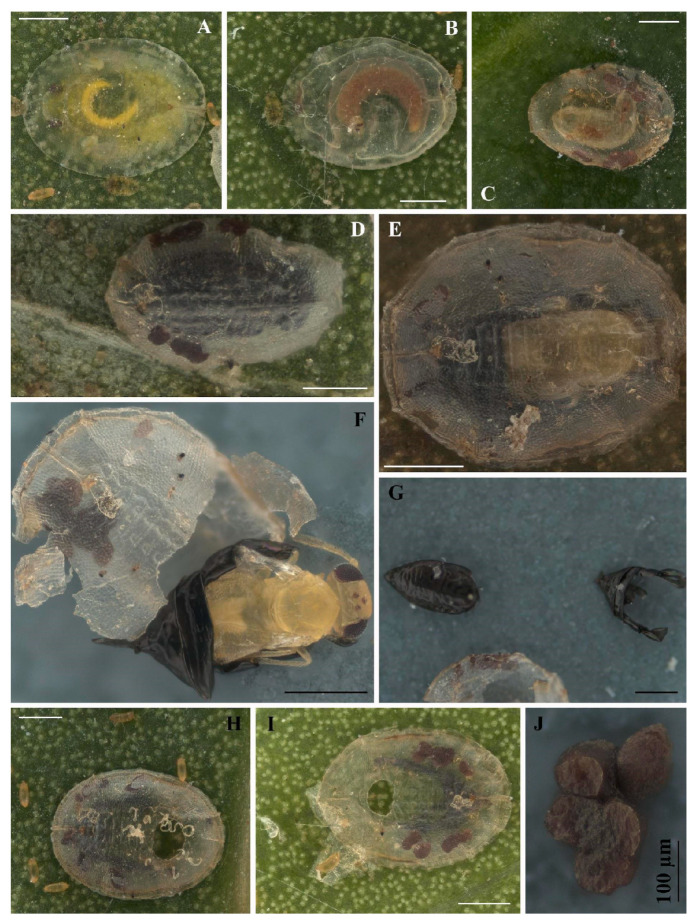
*Encarsia protransvena*: (**A**–**C**) larvae; (**D**) pupa; (**E**), pupa ready to emerge; (**F**,**G**) dissection showing the emerging adult and pupal remains; (**H**,**I**) puparia cases of *S. simplex* with *E. protransvena* emerging hole; (**J**) meconium of *E. protransvena* (scale bar: 300 µm, except in (**J**)).

**Figure 6:**
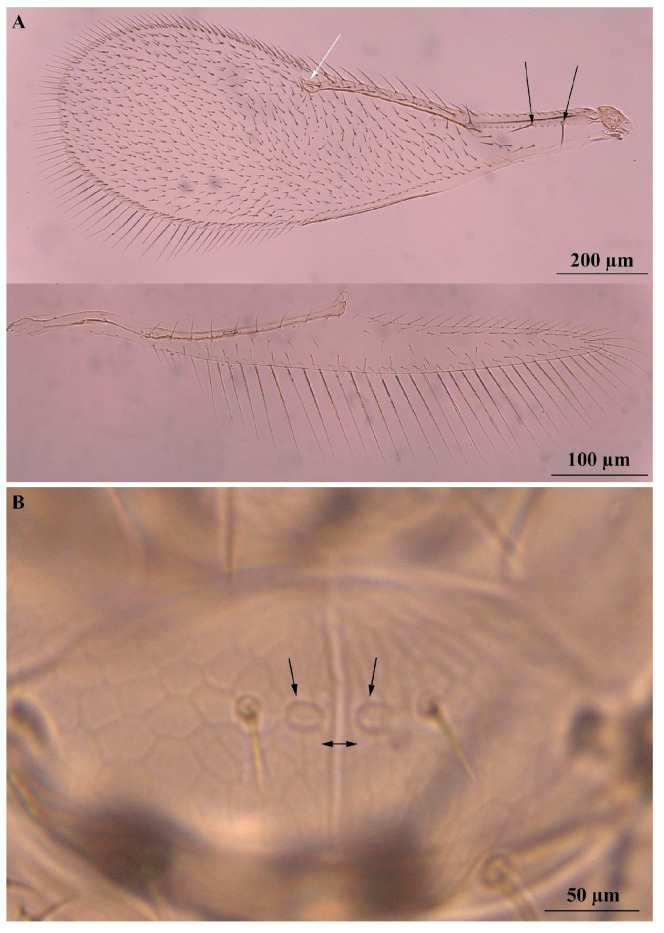
*Encarsia protransvena*: (**A**) forewing and hindwing, the black arrow indicating the presence of two large setae on the submarginal vein and the white arrow a bare area just above the stigmal vein; (**B**) scutellar sensillae (black arrows) closely placed or touching, ovoid and separated by less than their maximum diameter, rarely by a full diameter (left-right black arrow indicates the distance between the sensilla).

**Figure 7:**
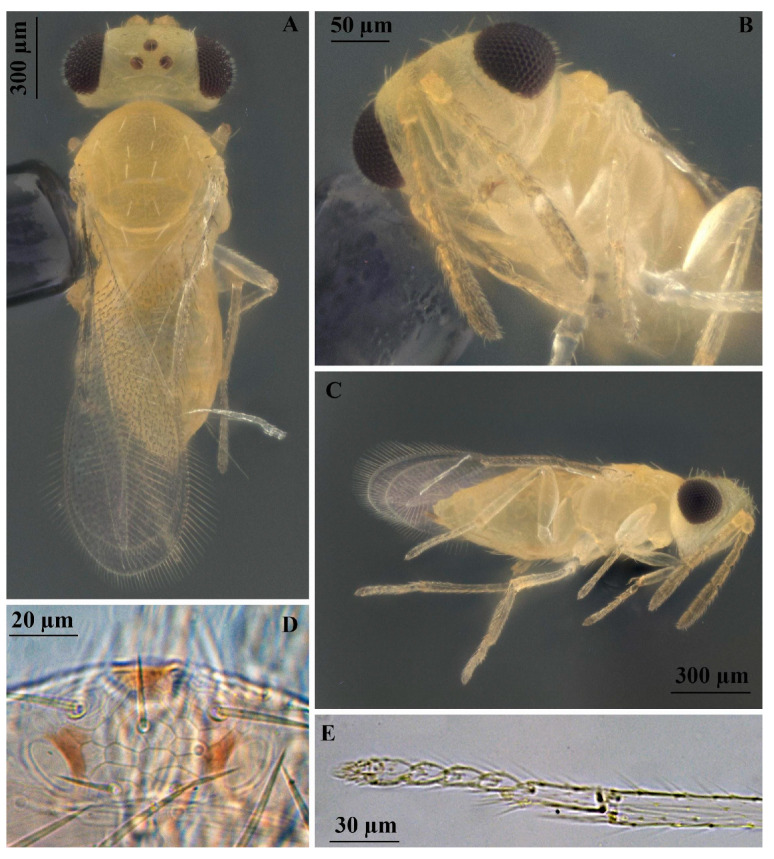
*Encarsia protransvena*: (**A**) body, dorsal view, mid-lobe of mesosoma with 4 pairs of setae, preapical pair not reaching the base of apical pair; (**B**) head, ventrolateral view; (**C**) body, lateral view; (**D**) stemmaticum, reticulate; (**E**) apex of middle tibia and tarsus.

**Figure 8:**
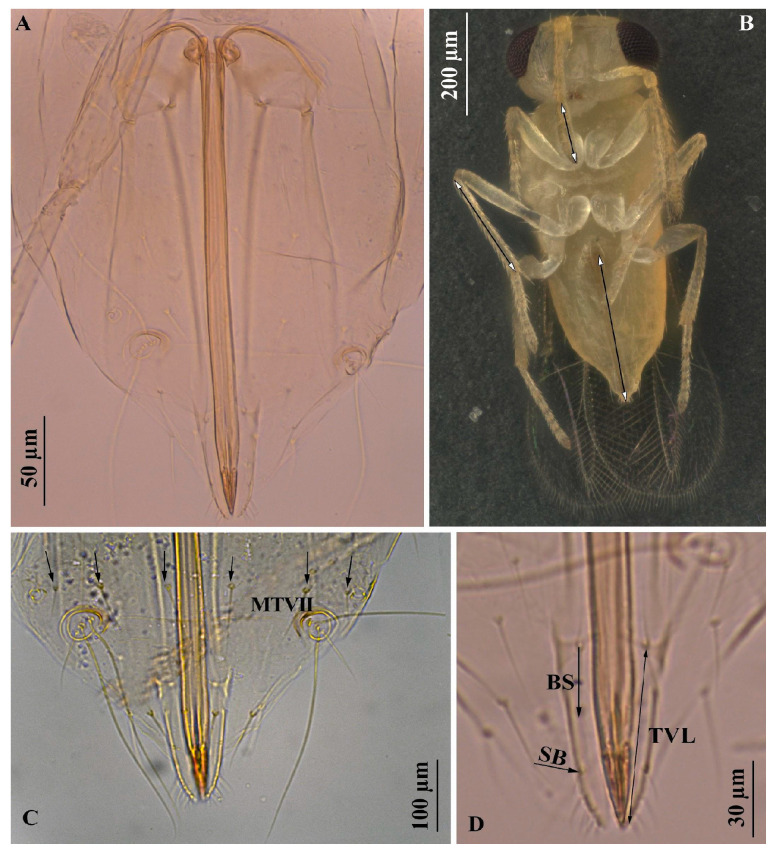
*Encarsia protransvena*: (**A**) ovipositor slender and with straight tip; (**B**) body, ventral view, the arrows indicate ovipositor, antennal clava and middle tibia length; (**C**) seventh metasomal tergum (Mt7) with 6 setae, 4 long setae medial to cerci; (**D**) basal seta (BS) of third valvula (TVL) not reaching base of subapical seta (SB); subapical seta located beyond halfway (0.65) between basal seta of third valvula and apex.

***Encarsia protransvena*** Viggiani, 1985

(Figure 5, Figure 6, Figure 7 and Figure 8)

**Examined material.**

12 ♀♀ on slides, (ITALY: Sicily, Palermo, 9.II.2024, ex *S. simplex* (Singh, 1931) on *F. microcarpa* L. f., leg. G. Cerasa). 9 ♀♀ on slides, (ITALY: Sicily, Palermo, 16.II.2024, ex *S. simplex* (Singh, 1931) on *F. microcarpa* L. f., leg. L. Tomasello) (Department of Agricultural, Food and Forest Sciences, University of Palermo).

20 ♀♀ on slides, (ITALY: Calabria, Reggio Calabria, 7.X.2024, ex *S. simplex* (Singh, 1931) on *F. microcarpa* L. f., leg. V. Palmeri) (Department of Agriculture-Mediterranean University of Reggio Calabria).

2 ♀♀ in ethanol (ITALY: Sicily, Catania, 6.X.2024, ex *S. simplex* (Singh, 1931) on *F. microcarpa* L. f., leg. G. Siscaro). 10 ♀♀ on slides and 30 ♀♀ in ethanol (ITALY: Sicily, Acicastello (CT), 12.X.2024, ex *S. simplex* (Singh, 1931) on *F. microcarpa* L. f., leg. G. Siscaro). 10 ♀♀ in ethanol (ITALY: Sicily, Acireale (CT), 7.X.2024, ex *S. simplex* (Singh, 1931) on *F. microcarpa* L. f., leg. G. Siscaro) (Department of Agriculture, Food and Environment, University of Catania).

10 ♀♀ on slides and 11 ♀♀ in ethanol (ITALY: Sicily, Palermo, 9.II.2024, ex *S. simplex* (Singh, 1931) on *F. microcarpa* L. f., leg. G. Cerasa). 3 ♀♀ in ethanol (ITALY: Campania, Santa Maria Capua Vetere, 30.IX.2024, ex *S. simplex* (Singh, 1931) on *F. microcarpa* L. f., leg. P. A. Pedata). 5 ♀♀ in ethanol (ITALY: Lazio, Formia, 5.X.2024, ex *S. simplex* (Singh, 1931) on *F. benjamina* L., leg. P. A. Pedata). 2 ♀ in ethanol (ITALY, Campania, Naples, 27.XI.2024, ex *S. simplex* (Singh, 1931) on *F. benjamina* L. f., leg. P. A. Pedata) (Department of Agricultural Sciences, University of Naples Federico II).

**Original description:** Viggiani [39] described the species based on 1 ♀, holotype ex *Dialeurodes kirkaldyi* (Kotinsky, 1907) collected in U.S.A, Florida, Sept. ’84, leg. C.R.R. Thomson; 6 ♀, paratypes, same data.

**Distribution:** Argentina, China, Colombia, Egypt, French Polynesia, Georgia, Honduras, Indonesia, Iran, Italy, Malaysia, Mexico, Puerto Rico, Spain, Taiwan, Turkey, USA, Western Australia [24,27,49]. We first report *E. protransvena* associated with the invasive whitefly *S. simplex* in Italy.

**Hosts:** The known hosts of *E. protransvena* are *Acaudaleyrodes citri* (Priesner et Hosny, 1934) [50], *Aleurotrachelus rubi* Takahashi, 1933, *Aleurothrixus floccosus* (Maskell, 1896), *Bemisia porteri* Corbett, 1935 [51], *B. tabaci* (Gennadius, 1889) [40,50,52,53], *Dialeurodes citri* (Ashmead, 1885) [40,53,54], *D. citrifolii* (Morgan, 1893) [40,50,52,53], *D. kirkaldyi* (Kotinsky, 1907) [39,40,50,52,53], *Parabemisia myricae* (Kuwana, 1927) [40,55,56], *Singhiella citrifolii* (Morgan, 1893) [56], *S. simplex* [24], *Tetraleurodes acaciae* (Quintance, 1900) [57], *Trialeurodes abutiloneus* (Haldeman, 1850) [40], *T. packardi* (Morrill, 1903) [40,50,53,58], *T. vaporariorum* Westwood, 1856 [52,54], *T. variabilis* (Quaintance, 1900) [40].

**Diagnosis:** The species has been included in the *strenua*-group [59], which includes about 40 species [40]. The authors previously mentioned redescribed *E. protransvena*, *E. citri* (Ishii, 1938), and *E. strenua* (Silvestri, 1927), adding two new species to the *strenua*-group: *E. neocala* Heraty and Polaszek, 2000 and *E. bimaculata* Heraty and Polaszek, 2000. The authors stressed that univariate and bivariate measures of morphological characters could not distinguish all five species because their ranges overlapped. However, by performing a multivariate morphometric analysis, all species could be discriminated. The authors then developed an identification key for females of the *strenua-*group species. Furthermore, they described the males of all these species except *E. neocala* but did not use them in the separation of the species, probably because their differences were minimal and sometimes the males are scarce, as for the thelytokous species *E. protransvena* (2 males out of 360 females examined) [55].

Members of the *strenua*-group are identified by the combination of two–three marginal setae along the dorsal margin of the costal cell at the apex, a bare area just above the stigmal vein, and scutellar sensillae that are closely placed or touching (Figure 6A,B). According to Heraty and Polaszek [40], within the *strenua*-group, *E. protransvena* is distinguished from closely related species of the same group by the following characteristics: mid-lobe of mesosoma usually with four pairs of setae, preapical pair not reaching the base of apical pair (Figure 7A); forewing (Figure 6A) usually more than 2.7 times as long as broad, if between 2.6 and 2.7 times, antennal club less than 0.14 mm in dorsal length; ovipositor slender with tip always straight (Figure 8A), clearly shorter than the length of gaster, less than 1.5 times as long as the middle tibia and more than 2.0 times as long as club (Figure 8B); apex of gaster (tergite 7 with spiracles) with six setae, four long setae medial to cerci (Figure 8C); basal seta of third valvula not reaching base of subapical seta, subapical seta located beyond halfway (0.65) between basal seta of third valvula and apex (Figure 8D).

***Encarsia hispida*** De Santis, 1948

(Figure 9)

**Examined material.**

1 ♀ on slide and 1 ♀ in ethanol (ITALY, Campania, Naples, 27.XI.2024, ex *S. simplex* (Singh, 1931) on *F. benjamina* L. f., leg. P. A. Pedata) (Department of Agricultural Sciences, University of Naples Federico II).

**Original description:** De Santis [60] described the species based on 1 ♀, holotype ex an unidentified aleyrodid on *Salvia splendens* (Schultes, 1820) [s/aleirodoideo en coral rojo] collected in Argentina, Santa Fe, Rosario, 1947 leg. Hack; 2 ♀, paratypes, same data.

**Distribution:** Argentina, Barbados, Brazil, Chile, Colombia, Dominican Republic, France (Guadeloupe, mainland), Guatemala, Honduras, Italy, Jamaica, Mexico, Netherlands (indoors), Peru, Portugal (Madeira), Puerto Rico, South Africa, Spain (Canary Islands, mainland), U.S.A., Venezuela [41].

**Hosts:***Aleurodicus dugesii* Cockerell 1896, *Aleuroglandulus subtilis* Bondar 1923 [=*A. malangae* Russell 1944], *A. floccosus*, *Aleurothrixus porteri* Quaintance and Baker 1916, *A. rhamnicola*, *Aleurotrachelus trachoides* (Back 1912), *Aleyrodes spiraeoides* Quaintance 1900, *B. tabaci*, *Dialeurodes* sp., *Lipaleyrodes* sp., *Metaleurodicus minimus* (Quaintance 1900), *P. myricae* [41], *S. simplex* [17,27,29], *S. phillyreae*, *T. acaciae, T. abutiloneus*, *Trialeurodes floridensis* (Quaintance 1900), *T. vaporariorum*, *T. variabilis* (Quaintance 1900) [41].

**Diagnosis:** The species belongs to the *luteola*-group, which is characterized by four-segmented mid tarsus, fore wing without an asetose area around the stigmal vein, antennal formula 1:1:4:2 [61,62]. Polaszek et al. [41] included it in the *E. meritoria* species-complex of the *luteola* group, together with three more species (*E. dispersa* Polaszek, *E. haitiensis* Dozier and *E. meritoria* Gahan). They are all characterized by the typical female body color, generally mostly yellow, and by the tibial spur of the midleg, only slightly shorter than the corresponding basitarsus. Based on this latter character and molecular analysis of the 28S rDNA D2 region, a fifth yellow species, *E. californica* Polaszek, was not included in the species-complex and considered more closely related to *E. formosa* Gahan and *E. luteola* Howard. The authors could separate these very similar species based on the proportion of funicular segments, since these differences, even though very subtle, were consistently supported by multivariate and molecular analysis.

**Figure 9:**
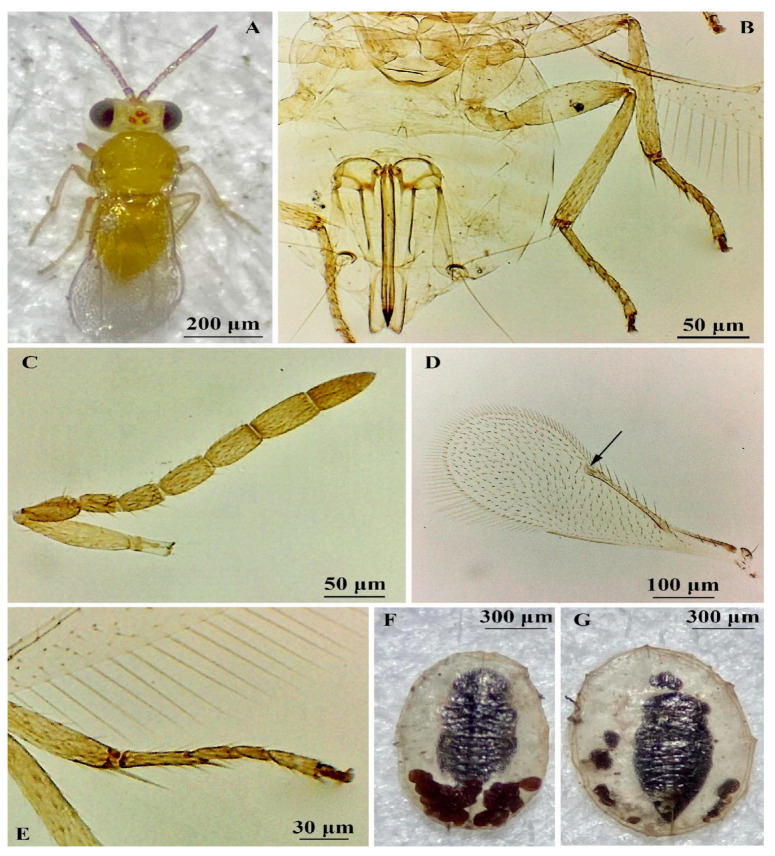
*Encarsia hispida* (**A**–**F**) and *Encarsia protransvena* (**G**): (**A**) adult female, dorsal view; (**B**) ovipositor, middle and hind tibia; (**C**) antenna; (**D**) forewing, the black arrow indicates the absence of a bare area around the stigmal vein; (**E**) apex of middle tibia, showing mid tarsus segments and the spur-to-basitarsus relative length; (**F**) pupa of *E. hispida* and (**G**) pupa of *E. protransvena*.

According to Polaszek et al. [41], the female of *E. hispida* can be separated from the other members of the *meritoria* complex by the following combination of characters: relative length of funicular segments, with F2 intermediate between F1 and F3; F6 1.2× as long as F5; scape less than 1.6× as long as F6; pedicel elongate, 1.2–1.3× as long as F1, mid tibial spur 0.75–0.8× as long as corresponding basitarsus (Figure 9A–E). The most closely related species in the complex is *E. meritoria*, whose males have antennae with F5 and F6 partially fused, while males of *E. hispida* have F5 and F6 separated.

Due to these similarities, these two species have passed through a complex taxonomic history since, after the synonymy proposed by Viggiani [63], Polaszek et al. [64] revalidated *E. hispida*. Considering that *E. meritoria* is known only from two series, both collected in Miami Beach, Florida, in 1918 and 1927, the taxonomic status of the two entities will not be fully understood as long as fresh material of *E. meritoria* from type locality will be available for molecular and biological analysis.

Moreover, *E. hispida* was successively considered conspecific with *E. brasiliensis* (Hempel) [41]. The authors proposed to maintain the name of the former species with respect to the senior synonym by reversal of precedence, considering that the type material of *E. brasiliensis* has been lost and that, due to a misidentification of Dozier [65], the name *E. brasiliensis* has been consistently applied to a distant taxonomic species belonging to the *E. opulenta* group.

**Key to the females of *Encarsia* spp. reared from *S. simplex*:**

1.Antennal formula 1.1.3.3
*E. protransvena* (Figure 6, Figure 7 and Figure 8)--Antennal formula 1.1.4.22
2.Body entirely yellow; forewing hyaline; mid tarsi 4-segmented, tarsal formula 5-4-5

*E. hispida* (Figure 9)--Body yellow with brown areas on mesosoma and metasoma; forewing slightly infuscate below marginal vein; mid tarsi 5-segmented, tarsal formula 5-5-5 

*E. singhiellae*



### 3.2. Molecular Analysis

Two *E. protransvena* specimens collected in Palermo on 9.II.2024 and one *E. hispida* found in Naples on 27.XI.2024 (see Examined material) were further validated through sequence analysis of the mitochondrial *COI* locus and the nuclear *28S-D2* region. The expected sequences of parasitoids that emerged from *S. simplex* hosts were successfully amplified, sequenced, and uploaded to GenBank under accession numbers PQ451909 (*COI*) and PQ451935 (*28S-D2*) for *E. protransvena*, and PQ725685 (*COI*) and PQ725690 (*28S-D2*) for *E. hispida*.

The amplified *COI* portion and the *28S-D2* nuclear region were 774 and 598 bp long, respectively, in both parasitoids. The mitochondrial sequence of *E. protransvena* was truncated from its original size of 910 bp to exclude the non-transcribed region between the locus and the tRNA-leu gene, as previously described in molecular studies on *Encarsia* species [43].

For *E. protransvena*, the BLASTn analyses of the obtained *COI* sequences confirmed a complete match with the only sequence available in GenBank (AY264341.1) referring to specimens collected in Naples (Campania region, Italy) parasitizing *P. myricae* on *Citrus* spp. [43]. In addition, the *28S-D2* nuclear dataset showed 100% identity with the two sequences in the database (AF254209.1; AF254208.1) associated with Californian samples [61].

The identity of *E. hispida* was also confirmed, with its mitochondrial sequence perfectly aligning with a GenBank entry (EU488723.1) from specimens in Naples (Campania region, Italy) and San Diego (California) emerging from *Bemisia tabaci* Gennadius and *Aleurodicus dugesii* Cockerell hosts, respectively [66]. At the nuclear level, a single nucleotide substitution in the *28S-D2* region resulted in 99.83% similarity to Californian specimens reported in the NCBI database (AF223370.1). These mitochondrial and nuclear sequences provided strong genetic support for the molecular comparative identification of both *Encarsia* species associated with the fig whitefly.

## 4. Discussion

Since its first detection in Italy, *S. simplex* has caused significant damage to ornamental *Ficus* plants in several regions [14,15,16,17]. The insect was probably introduced through the trade of plants from infested areas, such as Southeast Asia [15]. Given the ornamental function of many *Ficus* plants and their location mainly in public places, where the application of pesticides is limited and problematic, it is essential to monitor the presence and effectiveness of natural enemies before implementing any management action, whether chemical or agronomic. In addition to the activities of predators, especially coccinellids, several A.A. report an average parasitization rate of whiteflies of 10% due to *Encarsia* spp. [22,29]. Although *B. balios* and *A. bennetti* appear to be the more effective parasitoids of *S. simplex* in the U.S.A. [6,28], *E. protransvena* provides a limited level of control in Turkey, where the highest natural parasitism rate was found to be 32.88% and 21.66% in October 2018 and 2019, respectively [24]. Preliminary data from sampling in the study site at Palermo (Sicily) from May 2024 confirm that the highest parasitism rate, about 20%, was recorded in October (unpublished data). The neotropical species *E. hispida* has been reported parasitizing *Aleurodicus dispersus* Russel, 1965 on *F. macrophylla* Pers. and *Ficus* spp. in the Canary Islands and Madeira [67]. This species, belonging to the *luteola*-group, was recently reported as a parasitoid of *S. simplex* in Northern Italy [17]. However, comparing the *E. hispida* description with the figures of the slide-mounted specimen reported, the identification appears to be doubtful. *Encarsia hispida*, as described by De Santis [60], is similar to *E. protransvena* in body color and easily distinguishable from the latter by the antennal and tarsal formulas (see the key to female species reported in the previous paragraph). The molecular characterization of *E. hispida* reported by Zugno et al. [17] and the slide image, clearly attributable to *E. protransvena,* suggest the possibility of a mixed population of the two parasitoids in the sampled area, as confirmed in Campania. Additionally, Zugno et al. [17] relied on *COI* sequences to support their identification of *E. hispida* but did not provide GenBank accession numbers for their data, instead directly using the BLASTn tool for sequence comparison, limiting reproducibility and broader comparative analyses. It is worth noting that these observations do not detract from the contributions of Zugno et al. [17] about the distribution of these parasitoids in Italy.

Integrating morphological and molecular analyses for the identification of parasitic Hymenoptera provides a robust and reliable method for enhancing taxonomic accuracy [68,69]. In this study, we applied a combined morphological and molecular approach to comprehensively identify *E. protransvena* and *E. hispida* using two informative molecular markers, *COI* and *28S*, corroborating our morphological findings. Molecular evidence derived from BLASTn analysis confirmed that the *E. protransvena* population we studied is genetically indistinguishable from the previously described in Italy [42] and California [61], as it shares identical mitochondrial and nuclear haplotypes.

This complete genetic identity among populations from geographically distant regions raises intriguing questions about dispersal mechanisms of this aphelinid wasp, highlighting the need for future investigation. Similarly, *E. hispida* showed high genetic homogeneity, with its mitochondrial sequence fully matching specimens from Italy and California [66]. A single nucleotide substitution in the *28S-D2* region indicated slight intraspecific nuclear variation (0.17% divergence), potentially reflecting regional adaptation or sequencing variability.

The high-resolution molecular dataset generated in this work represents a valuable resource for future research on the population genetics, phylogeography, and evolutionary dynamics of these aphelinid wasps, with important implications for ecological studies and potential biocontrol strategies against whiteflies.

Finally, the integrated taxonomy method allowed us to report for the first time in Italy the association of *E. protransvena* with the fig whitefly *S. simplex* and to confirm the association of *E. hispida*, already known in Italy, as a parasitoid of this invasive species.

## Figures and Tables

**Table 1:** Sampling sites, coordinates, host plants, and dates of *Singhiella simplex*, *Encarsia hispida* and *Encarsia protransvena* monitoring activities in central and south Italy.

Sampling Sites (Province)	Coordinates	Host Plant	Date
Palermo (PA)	38°06′20″ N; 13°20′57″ E	*F. microcarpa*	9.II.2024 16.II.2024
Catania (CT)	37°31′09″ N; 15°06′19″ E	*F. microcarpa*	6.X.2024
Acicastello (CT)	37°33′28″ N; 15°09′01″ E	*F. microcarpa*	12.X.2024
Acireale (CT)	37°37′44″ N; 15°09′33″ E	*F. microcarpa*	7.X.2024
Reggio Calabria (RC)	38°07′21″ N; 15°39′32″ E 38°07′13″ N; 15°39′29″ E 38°07′32″ N; 15°39′17″ E	*F. microcarpa*	7.X.2024
Santa Maria Capua Vetere (CE)	41°05′01″ N; 14°15′56″ E 41°04′42″ N; 14°15′42″ E	*F. microcarpa* *F. benjamina*	30.IX.2024 30.IX.2024
Salerno (SA)	40°40′38″ N; 14°45′53″ E 40°40′12″ N; 14°47′15″ E 40°38′33″ N; 14°51′26″ E	*F. benjamina*	25.X.2024 18.X.2024 8.XI.2024
Naples (NA)	40°51′35″ N; 14°16′14″ E	*F. benjamina*	27.XI.2024
Formia (LT)	41°15′48″ N; 13°37′33″ E	*F. benjamina*	5.X.2024
Bari (BA)	41°05′46″ N; 16°53′20″ E 41°06′31″ N; 16°52′23″ E	*F. microcarpa*	6.III.2024; 5.VI.2024; 15.VIII.2024; 17.IX.2024; 14.X.2024; 21–22.XI.2024 (in both sites, only *S. simplex*)

**Table 2:** Primers used for PCR analysis targeting the DNA barcoding regions of *Encarsia protransvena* and *Encarsia hispida* for molecular identification.

Name	Nucleotide Sequence (5′-3′)	Melt. Temp. (°C)	Target Gene
C1-J-2183 C1-J-2195 TL2-N-3014	CAACATTTATTTTGATTTTTTGG TTGATTTTTTGGTCATCCAGAAGT TCCAATGCACTAATCTGCCATATTA	55 55 63	*COI*
D2F D2R	AGTCGTGTTGCTTGATAGTGCAG TTGGTCCGTGTTTCAAGACGGG	57 62	*28S-D2*

## Data Availability

The original contributions presented in this study are included in the article. Further inquiries can be directed to the corresponding author(s).
